# Profiling intestinal microbiota of *Metaplax longipes* and *Helice japonica* and their co-occurrence relationships with habitat microbes

**DOI:** 10.1038/s41598-021-00810-9

**Published:** 2021-10-27

**Authors:** Haidong Li, Shanshan Li, Shiliang Fan, Yan Xu, Xiangli Tian

**Affiliations:** 1 School of Fishery, Zhejiang Ocean University, Zhoushan, 316022 China; 2 MNR Key Laboratory of Marine Eco-Environmental Science and Technology, First Institute of Oceanography, Ministry of Natural Resources, Qingdao, 266061 China; 3 Guangxi Key Lab of Mangrove Conservation and Utilization, Guangxi Mangrove Research Center, Beihai, 536000 China; 4 Key Laboratory of Mariculture, Ministry of Education, Ocean University of China, Qingdao, China

**Keywords:** Microbial communities, Molecular biology, Environmental sciences

## Abstract

Intestinal microbiota plays key roles in maintaining the health and homeostasis of the host. However, information about whether the formation of intestinal microbiota of wild aquatic animals is associated with habitat microbes is not fully understood. Here, intestine samples were collected from two wild crab species and sediment samples were collected from the habitat environment. The total DNA of each sample was extracted, and the V3–V4 regions of 16S rRNA were sequenced using the MiSeq platform. The purpose of this study was to investigate the composition and diversity of intestinal microbiota and habitat microbes, and bacterial community relationships between wild crab intestine and habitat sediment. In the present study, the composition and diversity of intestinal microbiota of the two crab species were different from the habitat microbes. In contrast, a similar composition and diversity of the intestinal microbiota were observed between two crab species. Moreover, the bacterial community relationships between crab intestine and habitat sediment were associated with intestinal regions. Further network analysis revealed that the network structure of the intestinal microbiota was not only associated with intestinal regions, but also with the crab species. Additionally, although the compositions of bacterial functions were similar between crab intestine and sediment, no significant correlation in bacterial functions was observed between crab intestine and sediment. The findings of the present study would contribute to understanding the relationship between intestinal microbiota of wild aquatic animal and habitat microbes, and providing new insights into the intestinal microbiota of wild aquatic animals.

## Introduction

Grapsid crabs are important components of macrobenthos in the intertidal zones^1^. Among them, species of *Helice* and *Metaplax* are common inhabitants of mangrove-fringed mudflats^1,2^. For example, *Metaplax longipes* and *Helice japonica* are widely distributed in mangrove-fringed mudflats. *M. longipes* inhabits especially in the high intertidal zone^3^, while *H. japonica* is found around the mid-intertidal zone^4^. As revealed that *M. longipes* and *H. japonica* are also widely distributed in Chinese coastal regions^3,5^, and both of them living in the same mudflats are observed in mangroves of Beibu gulf ^6^. Crabs actively dig and maintain burrows in the sediment, and the burrows affect sediment topography and biogeochemistry by modifying particle size distribution, drainage, redox conditions and organic matter as well as nutrient availability^7^. The ecological importance of crabs seems well recognized^8,9^. Kristensen^2^ provided a comprehensive review of grapsid crabs, describing them as ecosystem engineers, and they could affect the microbial and biogeochemical functional diversity in sediments. An increasing quantity of data now suggest that the relationship between the grapsids and mangroves is strongly reciprocal, with each influencing the performance or even survival of the other^1,10^. To sum up, the roles of the biology and ecology of grapsid crabs have been widely concerned by scholars. However, there has been less concerned about how habitat affects the symbiotic microbiome of crabs.

Based on the current knowledge on the intestinal microbiota, it seems obvious that their activities are closely associated with the health of the host^11–13^. However, intestinal microbiota is highly malleable^14^ and the shape of intestinal microbiota can be affected by diet, lifestyle, host genetics and other factors^13,15–17^. Sun et al.^18^ reported that dietary lipid levels could affect the composition of the intestinal microbiota in *Portunus trituberculatus*. Yang et al.^19^ reported that glyphosate significantly influenced intestinal microbial diversity of *Eriocheir sinensis*. Apine et al.^20^ reported that farming did not significantly alter the composition of the gut microbiome when compared to wild-caught crabs. It should also be noted that those studies have mainly focused on economically important crabs. By contrast, little is currently known about the intestinal microbiota of the wild crabs. In addition, recently, the relationship between bacterial community of habitat environment and intestinal microbiota of the host were reported. Fan et al.^21^ reported that bacterial compositions in shrimp intestine were similar to those in sediment no matter at freshwater or marine cultured environment. Huang et al.^22^ also found a closer relationship between microbiotas in sediment and shrimp intestine. The study of Dou et al.^23^ showed that the bacteria in sea cucumber intestine and culture pond were similar. However, information about the relationship between natural environmental microbes and intestinal microbiota of wild crabs remained unknown.

Previous study has confirmed that the composition and diversity of intestinal microbiota of middle-intestine were significantly different from the hind-intestine in the aquatic animal^24^, and the composition of intestinal microbiota in aquaculture animal was related to microbial community composition in sediment^21,25^. However, the bacterial composition of different parts of crab intestine and bacterial co-occurrence relationships between crab intestine and sediment remained unknown. In this study, *M. longipes and H. japonica* were collected in the same mudflats in Beihai Caotou village, and we used high-throughput sequencing to investigate the bacterial communities between crab intestine and intertidal sediment. We hypothesized, (1) the form of intestinal microbiota was related to crab species; (2) the bacterial co-occurrence relationships between crab intestine and sediment were associated with intestinal regions; (3) the bacterial community composition in middle-intestine was different from the hind-intestine.

## Results

### Sequencing statistics and shared OTUs

**Figure 1:**
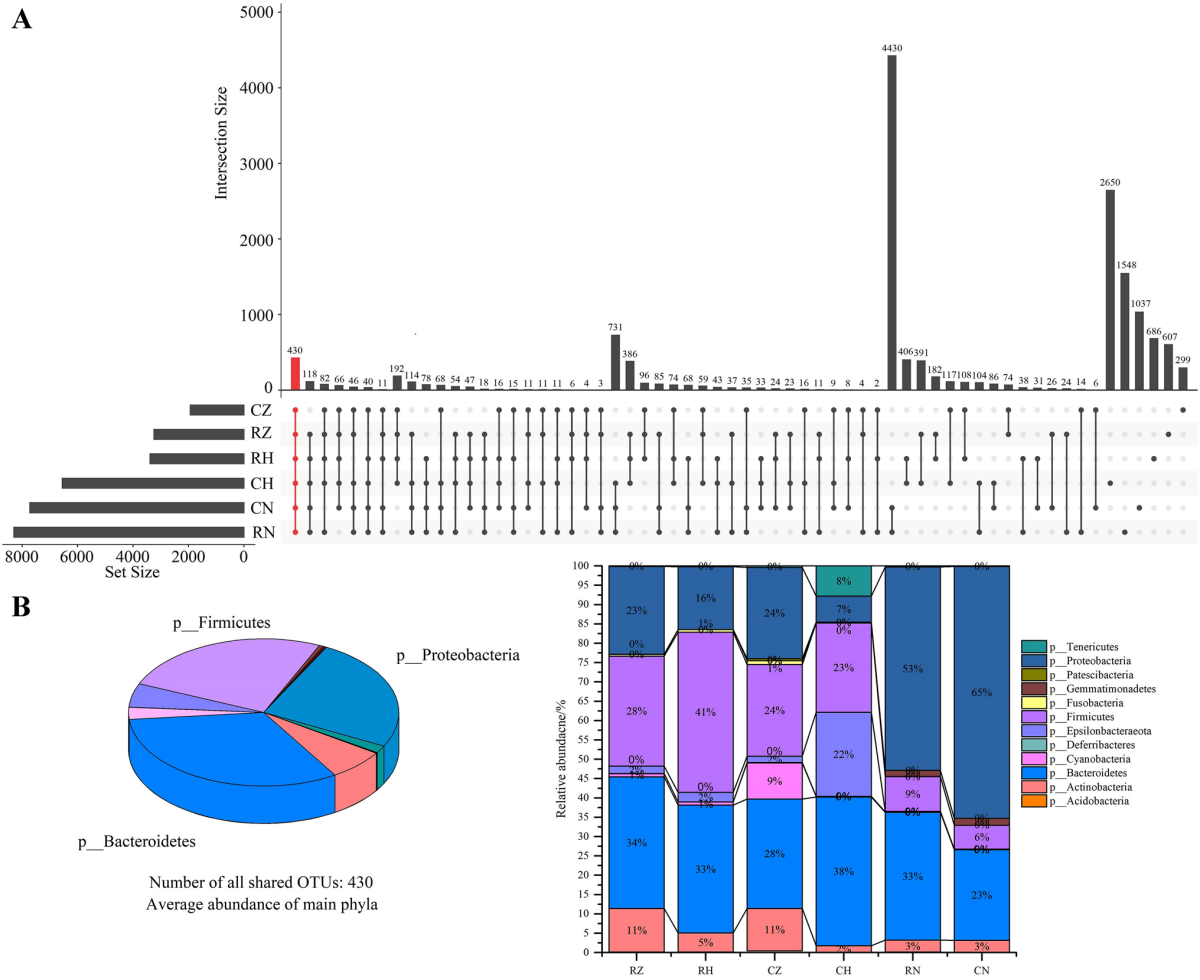
UpSet plot analysis at the operational taxonomic unit (OTU) level in all groups (**A**) and the distribution of shared OTUs at phylum level in all groups (**B**).

In total of 2,742,624 (max: 84,195, min: 62,837, mean: 76,184, SD =  ± 4624.31) valid tags were presented among all samples. The sequencing data of all samples were shown in the Table S1. The Good’s estimated sample coverage (ESC) index was 98.69–99.81%, indicating that the current sequencing depth could sufficiently represent the real situation of each sample. As shown in Fig. 1A,B, 430 OTUs were shared among all groups, and most shared OTUs were affiliated to Bacteroidetes, Firmicutes and Proteobacteria. CH group got the most unique OTUs, followed by RN, CN, RH, RZ and CZ groups.

### α- and β-diversity

**Figure 2:**
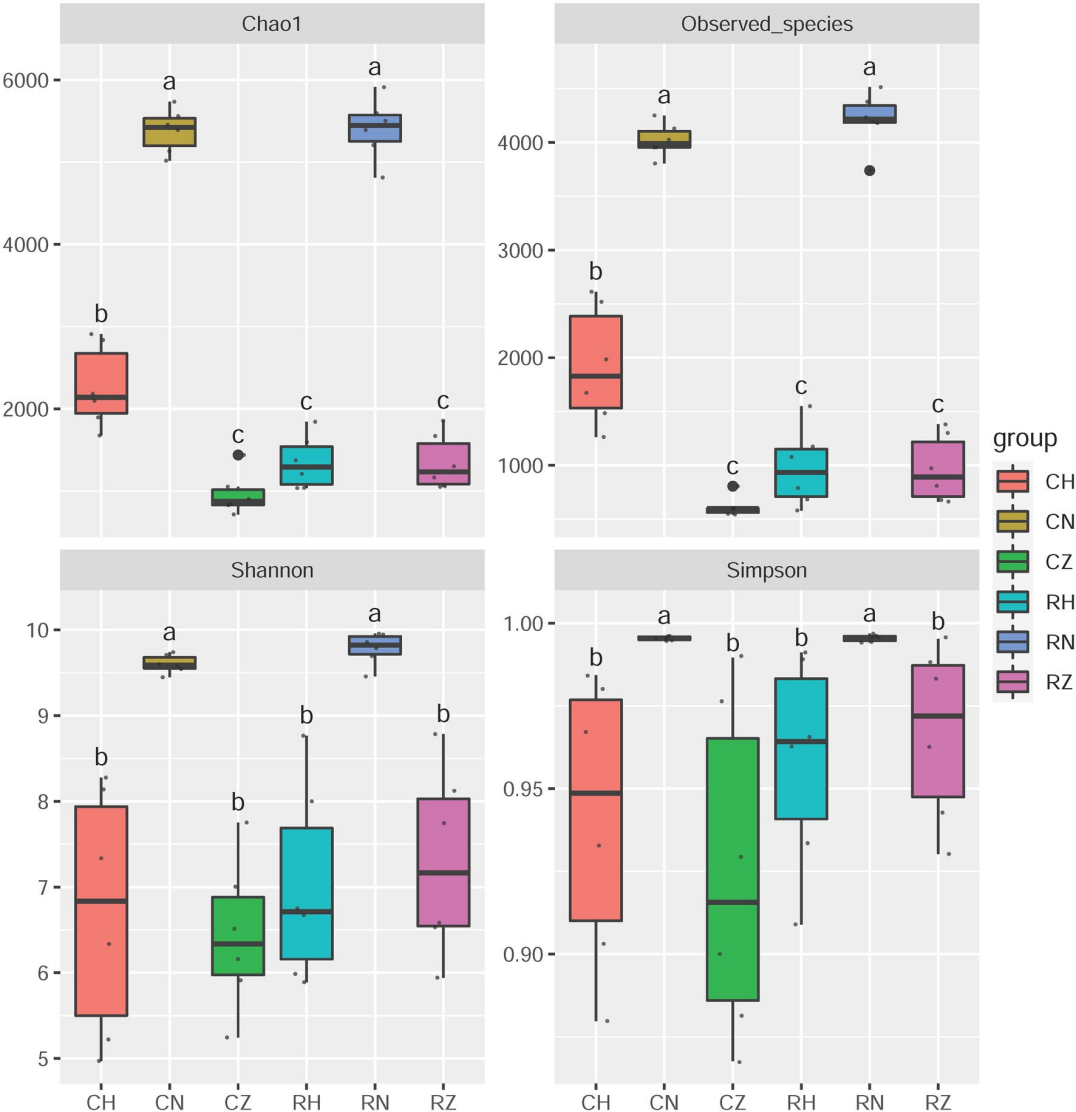
α-diversity analysis in all groups. Data with different letters at the column indicate significant differences (*P* < 0.05).

The complexity of each group was evaluated by Chao1, Observed_species, Simpson and Shannon indices. As shown in Fig. 2, these four indices in CN and RN groups were significantly higher than those in the other groups, indicating that the diversity and richness of microbial community in habitat sediment were significantly higher than those in crab intestine. The diversity of intestinal microbiota in terms of Simpson and Shannon indices had no significant difference among CZ, CH, RZ and RH groups (*P* > 0.05), indicating that the diversity of intestinal microbiota in middle-intestine had no significant difference with that in hind-intestine. The richness of intestinal microbiota in CH group increased significantly in comparison with that in CZ group (*P* < 0.05), while no significant difference in richness of intestinal microbiota was observed between RZ and RH groups (*P* > 0.05).

**Figure 3:**
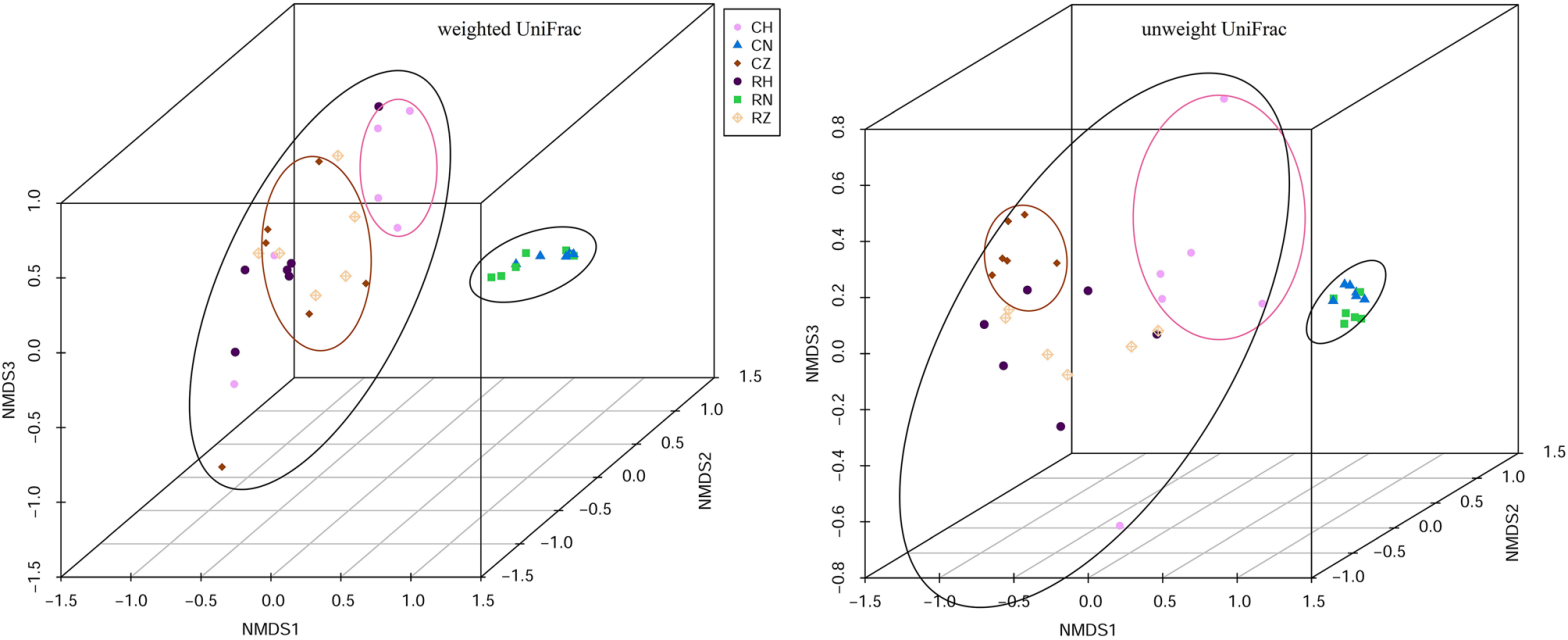
Non-metric multi-dimensional scaling analysis of all groups based on weighted and unweighted UniFrac metrics.

To explore the structure variance of microbial community among all groups, β-diversity were analyzed. The NMDS (non-metric multidimensional scaling) analysis showed that microbial communities belonging to the same group were more closely clustered with each other (Fig. 3). The microbial community in CZ, CH, RZ and RH groups were completely separated from that in habitat sediment, and the microbial community in CZ group were distinctly separated from that in CH group. Dissimilarity test was applied to explore the difference of microbial community structure between two groups (Table S2). PERMANOVA, ANOSIM and MRPP tests based on Bray–Curtis distance matrix revealed significant difference in microbial community structure between crab intestine and habitat sediment (*P* < 0.05). In addition, significant difference in microbial community structure was observed between CZ and CH groups (*P* < 0.05), while no significant difference in microbial community structure was found between RZ and RH groups (*P* > 0.05).

### Dominant composition and difference of the bacterial community

**Figure 4:**
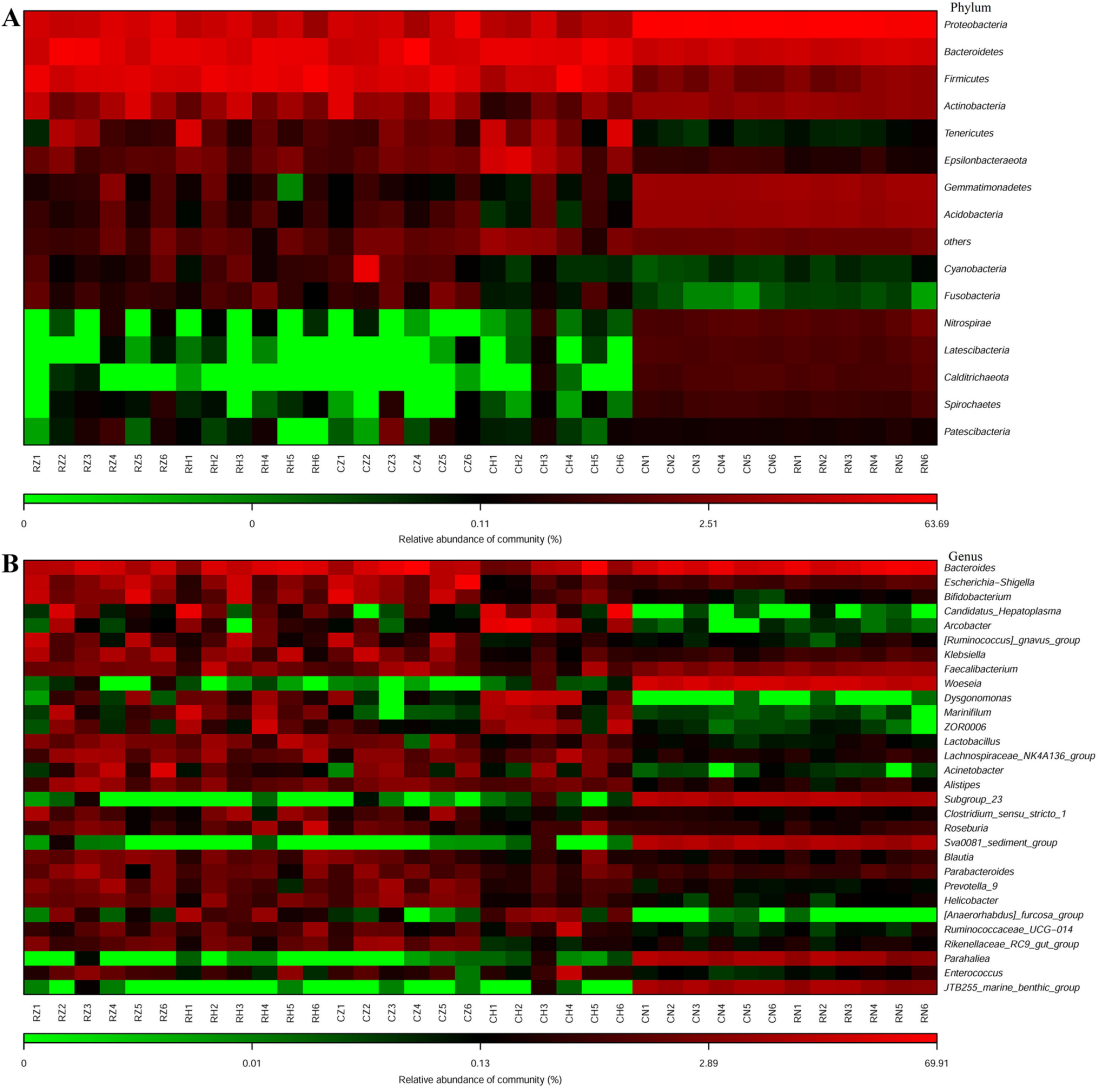
The composition of all groups at phylum (**A**) and genus level (**B**).

The 16S rRNA sequences in RZ, RH, CZ, CH, CN and RN groups were classified into 23, 30, 26, 24, 44 and 44 phyla, respectively. As shown in Fig. 4A and Table S3, Actinobacteria, Bacteroidetes, Firmicutes and Proteobacteria were the dominant phyla in RZ, RH and CZ groups, while the dominant phyla in CH group were Bacteroidetes, Epsilonbacteraeota, Firmicutes and Proteobacteria. No significant difference among dominant phyla were observed between RZ and RH groups (*P* > 0.05). The relative abundance of Epsilonbacteraeota of the CH group was significantly higher than that of the CZ group (*P* < 0.05), whereas the relative abundance of Proteobacteria of the CH group was significantly lower than that of the CZ group (*P* < 0.05). The relative abundances of Bacteroidetes and Firmicutes between CZ and CH groups were statistically the same. In CN and RN groups, the dominant phyla were Acidobacteria, Bacteroidetes, Gemmatimonadetes and Proteobacteria. No significant difference among dominant phyla were presented between CN and RN groups (*P* > 0.05). The relative abundances of Bacteroidetes and Firmicutes in the RZ, RH, CZ and CH groups were significantly higher than those in the CN and RN groups (*P* < 0.05). Whereas the relative abundances of Acidobacteria and Proteobacteria in the RZ, RH, CZ and CH groups were significantly lower than those in the CN and RN groups (*P* < 0.05). In addition, the relative abundance of Bacteroidetes, Firmicutes and Proteobacteria were statistically the same between RZ and CZ groups (*P* > 0.05), between RH and CH groups (*P* > 0.05).

At the genus level, as shown in Fig. 4B and Table S4, *Bacteroides*, *Bifidobacterium*, *Escherichia*-Shigella and [*Ruminococcus*]_gnavus_group dominated in intestinal microbiota of RZ and CZ groups, except for unidentified species. And the relative abundances of these four genera were statistically the same between RZ and CZ groups (*P* > 005). The dominant genera were *Bacteroides* and *Woeseia* in CN and RN groups, except unidentified species; and the relative abundance of *Woeseia* in CN and RN groups was significantly higher than that in RZ, RH, CZ and CH groups (*P* < 0.05). In CH group, the dominant genera were *Arcobacter*, *Bacteroides* and Candidatus*_Hepatoplasma*. Bacteroides and Candidatus*_Hepatoplasma* dominated in RH group. The relative abundances of *Bacteroides*, *Bifidobacterium*, Candidatus*_Hepatoplasma* and [*Ruminococcus*]_gnavus_group were statistically the same among RZ, RH, CZ, CH, CN and RN groups (*P* > 0.05), while the relative abundance of *Arcobacter* in CH group was significantly higher than that in RZ, RH, CZ, CN and RN groups (*P* < 0.05). In addition, the relative abundances of the potential beneficial bacteria *Bacillus*, *Bifidobacterium* and *Enterococcus* were not significant difference among all groups (*P > 0.05*); the relative abundance of *Lactobacillus* in RH group was significantly higher than that in CN and RN groups (*P* *< 0.05*), and the relative abundance of *Lactobacillus* among RZ, CZ, CH, CN and RN groups were statistically the same (*P* > 0.05). The relative abundance of pathogenic bacteria *Vibrio* was not significantly different among all groups (*P* > 0.05); the relative abundance of *Desulfovibrio* in RZ, CZ and CH groups were significantly higher than that in RH, CN and RN groups (*P* < 0.05); the relative abundance of *Escherichia*-Shigella in CZ group was significantly higher than that in CN and RN groups (*P* < 0.05), and the relative abundance of *Escherichia*-Shigella among RZ, RH, CH, CN and RN groups were statistically the same (*P* > 0.05) (Table S4).

### Classification of the bacterial taxa

The bacterial taxa of overall OTUs were showed in the Table 1. Most OTUs were assigned to RT and CRT in the RZ, RH, CZ, CH, CN and RN groups, which accounted for over 90% of overall OTUs. In the intestinal microbiota, the proportion of CRT was the highest in the RZ, RH and CZ groups (2.05, 1.59 and 1.91-fold of the RT, respectively), except for CH group (0.75-fold of the RT). While the proportion of RT in CN and RN groups was the highest (5.86 and 3.34-fold of the CRT, respectively). In addition, Bacteroidia and Clostridia were dominant in the RT and CRT of the RZ, RH, CZ and CH groups. In CN and RN groups, the dominant classes were Deltaproteobacteria and Gammaproteobacteria (Table S5). These results indicated that the composition of the bacterial taxa in the intestinal microbiota was different from that in the sedimental microbes.

**Table 1:** Categories of bacterial taxa.

Categories	RZ	RH	CZ	CH	CN	RN
Rare taxa, RT/%	32.03 (1095)	37.84 (1347)	33.48 (701)	56.22 (3799)	77.82 (6452)	72.74 (6414)
Abundant taxa, AT/%	0.00 (0)	0.00 (0)	0.05 (1)	0.00 (0)	0.06 (5)	0.03 (3)
Moderate taxa, MT/%	0.82 (28)	0.51 (18)	0.72 (15)	0.84 (57)	8.77 (727)	7.73 (682)
Conditionally rare taxa, CRT/%	65.69 (2246)	60.34 (2148)	63.90 (1338)	42.15 (2848)	13.29 (1102)	19.46 (1713)
Conditionally abundant taxa, CAT/%	0.47 (16)	0.53 (19)	0.72 (15)	0.31 (21)	0.06 (5)	0.07 (6)
Conditionally rare or abundant taxa, CRAT/%	0.99 (34)	0.84 (30)	1.15 (24)	0.47 (32)	0.00 (0)	0.00 (0)

The number of OTUs were showed in the bracket.

### Co-occurrence associations of bacterial community among different groups

**Figure 5:**
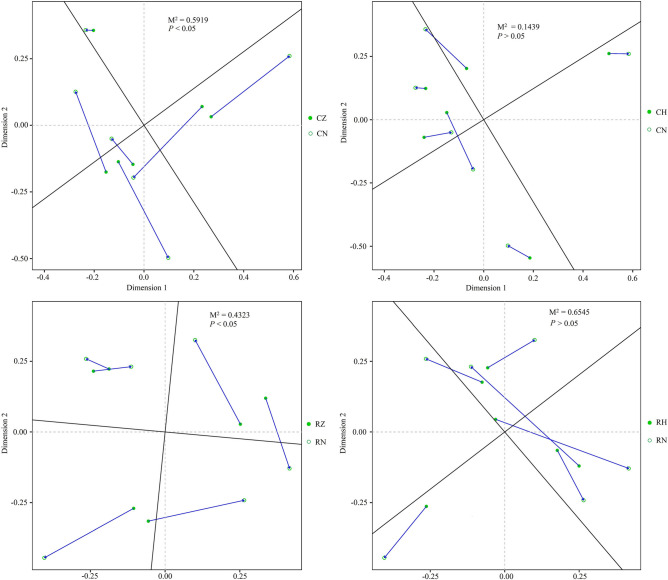
Procrustes analysis of bacterial community among different groups based on Bray–Curtis dissimilarity metrics.

The Procrustes test and Mantel test were performed to explore the relationship between bacterial community of crab intestine and the corresponding habitat bacterial community. As shown in Fig. 5, significant correlation of bacterial community was observed between CZ and CN groups (M^2^ = 0.5919, *P* < 0.05, 999 permutations), between RZ and RN groups (M^2^ = 0.4323, *P* < 0.05, 999 permutations). While no significant correlation in bacterial community was observed between CH and CN groups (M^2^ = 0.1439, *P* > 0.05, 999 permutations), between RH and RN groups (M^2^ = 0.6545, *P* > 0.05, 999 permutations). Mantel tests also showed that habitat bacterial community was the important factor for shaping the bacterial community in CZ and RZ groups (CZ vs CN, Spearman’s r = 0.5393, *P* < 0.05; RZ vs RN, Spearman’s r = 0.5036, *P* < 0.05) (Table S6).

### Network analysis and topological roles

In order to investigate the species interactions of the intestinal microbiota in RZ, RH, CZ and CH groups, pMENs (Phylogenetic molecular networks) were constructed. As shown in Table S7, CH network obtained the highest average connectivity (*avg*K), indicating that CH network was more complex than the other three networks. The average path length (GD) in CH network was significantly different from the other three networks (*P* < 0.05) and showed typical small-world network properties. GD was significantly different among four networks, indicating the structure of microbial communities in these four groups were obviously different. The modularity values in four networks varied from 0.773 to 0.879, indicating that the networks in four groups were naturally divided into modules. For the network system efficiency and robustness, average connectivity, average clustering coefficient, average geodesic distance and modules numbers were the important global properties of network, thus RZ network was more efficient and stable than RH network, and CH network was more efficient and stable than CZ network.

**Figure 6:**
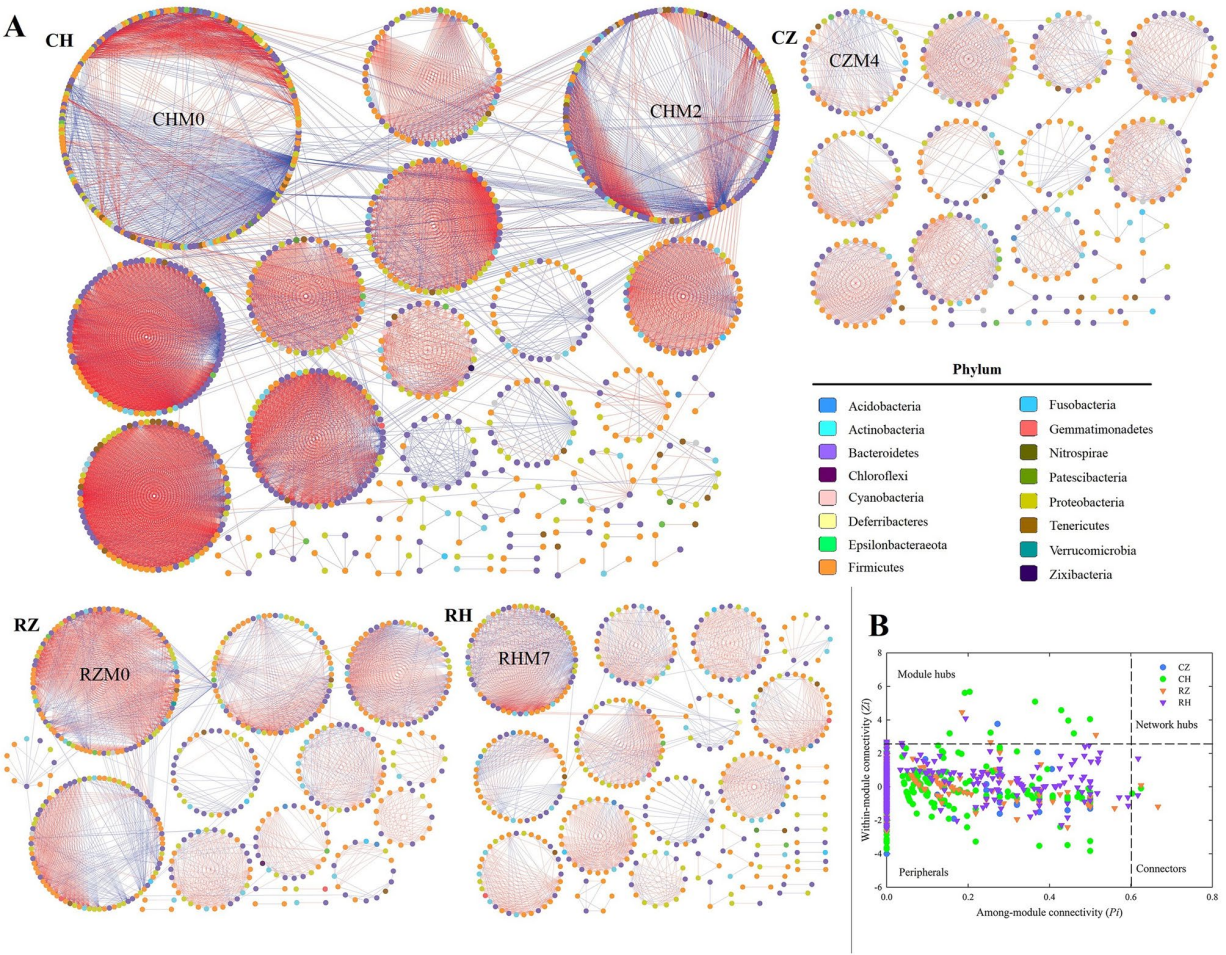
Ecological networks of intestinal microbiota in the six groups (**A**). Ecological network graph with submodule structure was obtained using the fast-greedy modularity optimization method. Each node indicates one OTU at the phylum level. Node colors indicate different major phyla. Blue edges indicate a positive interaction between two individual nodes, while red edges indicate a negative interaction. Z-P plot showing the distribution of operational taxonomic units (OTUs) based on their topological roles (**B**). Species with both a low Zi (value < 2.5) and a low Pi (value < 0.6) were peripheral species, i.e., they had only a few links, and these were almost always only to species within their module. Species with either a high value of Zi (≥ 2.5) or Pi (≥ 0.6) were module hubs, connectors and network hubs, as they were highly connected species linked to many other species within their own module.

The networks of RZ, RH, CZ and CH groups were visualized by Cytoscape software (Fig. 6A) and the composition of four networks were further analyzed and showed in Table S8. The red edges represented negative interactions between two species and the blue edges represented positive interactions between two species. RZ network had 17 sub-modules with 487 nodes and 3235 edges; the largest sub-module was RZM0 (99 nodes). RH network consisted of 35 sub-modules with 427 nodes and 2199 edges; the largest sub-module was RHM7 with 59 nodes. In CZ group, 28 sub-modules with 344 nodes and 1523 edges were observed in the network; the largest sub-module was CZM4 with 40 nodes. CH network had 40 sub-modules with 1036 nodes and 9761 edges; the largest sub-modules were CHM0 (203 nodes) and CHM2 (161 nodes). In these four networks, > 80% nodes (OTUs) affiliated to Bacteroidetes, Firmicutes and Proteobacteria; and negative interactions between two species dominated the four networks. Moreover, many nodes from the same phylum were found to be clustered within one sub-module in the four networks.

The complex networks could be described by the node degree, the network had more nodes with higher node degree implied more complexity^26^. The nodes with high node degree (≥ 50) in the four networks were showed in the Table S9. As shown in Table S9, the number of nodes with node degree ≥ 50 were 2, 6, 0 and 50 in the networks of RZ, RH, CZ and CH, respectively. This also proved that CH network was more complex than the other three networks. In addition, most nodes with node degree ≥ 50 were found to be clustered within one sub-module in the four networks and belonged to Bacteroidetes, Firmicutes and Proteobacteria.

The topological roles of species were distinctly divided into four types, namely, peripherals, module hubs, connectors and network hubs. As shown in Fig. 6B, overall OTUs were assigned to three types: peripherals, module hubs and connectors. The majority of OTUs in the four networks belonged to peripherals and several OTUs served as module hubs and connectors. As shown in Table S10, the species of module hubs and connectors affiliated to Bacteroidetes, Firmicutes and Proteobacteria.

### Functional prediction of the microbiota

**Figure 7:**
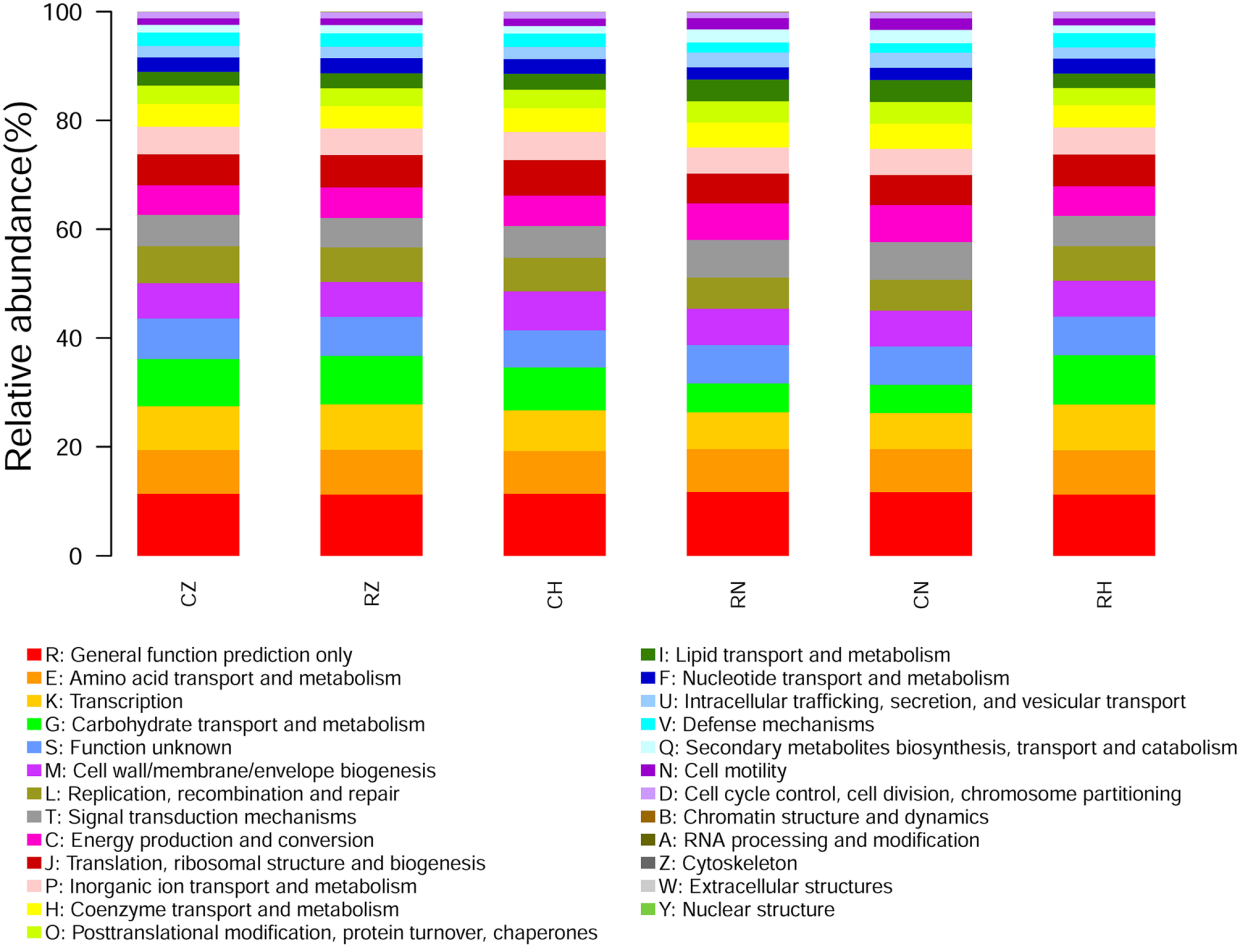
The COG function classification of the six groups.

The functional predictions of the microbiota of RZ, RH, CZ, CH, CN and RN groups were implemented using PICRUSt based on the eggnog database. As shown in Fig. 7, the functional profiles of all groups were much more like each other. The dominant functions in all groups were Amino acid transport and metabolism (RZ, 8.19 ± 0.12%; RH, 8.05 ± 0.15%; CZ, 7.99 ± 0.20%; CH, 7.80 ± 0.06%; CN, 7.81 ± 0.02%; RN, 7.78 ± 0.04%), Carbohydrate transport and metabolism (RZ, 8.71 ± 0.45%; RH, 8.87 ± 0.35%; CZ, 8.77 ± 0.53%; CH, 7.72 ± 0.46%; CN, 5.17 ± 0.06%; RN, 5.35 ± 0.09%), Cell wall/membrane/envelope biogenesis (RZ, 6.60 ± 0.26%; RH, 6.70 ± 0.22%; CZ, 6.64 ± 0.34%; CH, 7.19 ± 0.12%; CN, 6.62 ± 0.03%; RN, 6.67 ± 0.04%), Energy production and conversion (RZ, 5.62 ± 0.09%; RH, 5.42 ± 0.07%; CZ, 5.48 ± 0.10%; CH, 5.60 ± 0.14%; CN, 6.85 ± 0.02%; RN, 6.75 ± 0.04%),General function prediction only (RZ, 11.27 ± 0.10%; RH, 11.30 ± 0.07%; CZ, 11.45 ± 0.19%; CH, 11.42 ± 0.05%; CN, 11.74 ± 0.01%; RN, 11.78 ± 0.02%), Inorganic ion transport and metabolism (RZ, 4.88 ± 0.09%; RH, 4.97 ± 0.17%; CZ, 5.09 ± 0.17%; CH, 5.22 ± 0.17%; CN, 4.79 ± 0.01%; RN, 4.78 ± 0.02%), Replication, recombination and repair (RZ, 6.33 ± 2.65%; RH, 6.33 ± 2.44%; CZ, 6.77 ± 5.08%; CH, 6.15 ± 2.44%; CN, 5.66 ± 2.44%; RN, 5.72 ± 2.44%), Signal transduction mechanisms (RZ, 5.37 ± 0.08%; RH, 5.59 ± 0.17%; CZ, 5.64 ± 0.27%; CH, 5.85 ± 0.17%; CN, 6.90 ± 0.03%; RN, 6.91 ± 0.05%), Transcription (RZ, 8.19 ± 0.30%; RH, 8.33 ± 0.30%; CZ, 8.05 ± 0.30%; CH, 7.37 ± 0.35%; CN, 6.67 ± 0.02%; RN, 6.77 ± 0.04%), Translation, ribosomal structure and biogenesis (RZ, 6.10 ± 0.26%; RH, 5.95 ± 0.19%; CZ, 5.72 ± 0.14%; CH, 6.68 ± 0.30%; CN, 5.51 ± 0.01%; RN, 5.47 ± 0.02%). To explore the relationship between the bacterial community functions of crab intestine and the corresponding habitat bacterial community functions, Mantel test was performed. The results of the Mantel test showed that no significant relationship in functions of bacterial community were observed between crab intestine and habitat sediment (Table S11). In addition, the NMDS plot showed that the functional structure of bacterial community in crab intestine was completely separated from the functional structure of the corresponding habitat bacterial community (Fig. S1); and significant differences in bacterial functional structure were detected between crab intestine and the corresponding habitat sediment by ANOSIM test. In addition, similar results were also found in functional pathways of bacterial community between groups based on the Kyoto Encyclopedia of Genes and Genomes database, which could be acquired in Supplemental material (Fig. S2, S3 and Table S12).

## Discussion

The intestinal microbiota is closely related to the immune system functions and metabolic regulation of the host^27–29^. However, the intestinal microbiota is not conservative in the host, the composition and structure of it can be shaped by host species^16^, diet^15^, health^30,31^, age^32^ and the habitat environment^21,33,34^. In the present study, we tried to explore the composition of intestinal microbiota in different intestinal regions between two crab species, and the correlation in microbial community between crab intestine and habitat sediment.

Our data showed that the number of shared OTUs was a little between crab intestine and habitat sediment. This result was consistent with the report of Smith et al.^15^ who found that there had little shared OTUs between stickleback and water samples. Fan et al.^21^ reported that the diversity and richness of bacterial community in the sediments were significantly higher than those in the shrimp intestine, and the structure of bacterial community in the sediments was significantly different from the structure of intestinal microbiota of shrimp. Smith et al.^15^ also found that the structure of environmental microbiota was significantly different from the structure of intestinal microbiota of stickleback. In the present study, similar results were observed between bacterial community of habitat sediment and intestinal microbiota of wild crab. In addition, we also found significant difference in the richness and structure of the intestinal microbiota between the two crab species, while the diversity of the intestinal microbiota had no significant difference between the two crab species.

Previous studies showed that Actinobacteria, Bacteroidetes, Firmicutes and Proteobacteria were the abundant phylum in the intestine of aquatic animals, such as *Ctenopharyngodon idella*^24,35^, *Bostrichthys sinensis*^36^, *Ictalurus punctatus*^37^, *Apostichopus japonicus*^23,38^ and *Litopenaeus vannamei*^21,39–42^. It has been reported that Proteobacteria and Bacteroidetes are main bacteria taxon in sediment^23,25,42^, and they participate in various biogeochemical processes, such as carbon and nitrogen cycling^43,44^. Similar results were observed in this study. In addition, the relative abundances of dominant phyla between two crab species were different, suggesting that the compositions of abundant bacterial taxa could be shaped by host species.

Hou et al.^39^ reported that Actinobacteria, Bacteroidetes, Firmicutes, Gemmatimonadetes and Proteobacteria were the dominant phyla in the intestinal microbiota of *Litopenaeus vannamei*; and the relative abundance of those phyla between shrimp intestine and the sediment were significantly different. Fan et al.^21^ also found that the relative abundances of dominant phyla between shrimp intestine and sediment were significantly different. In present study, the most dominant phylum was Bacteroidetes in crab intestine, while Proteobacteria was the most dominant phylum in sediment; and the relative abundances of Bacteroidetes and Proteobacteria between crab intestine and sediment were significantly different. Firmicutes was the other dominant phylum in crab intestine, and its relative abundance in crab intestine was significantly higher than that in sediment. Thus, although the compositions of dominant phyla in crab intestine were similar with sediment, their relative abundances between intestine and sediment were significantly different. Meanwhile, the results of mantel test and Procrustes analysis showed that a closer relationship between microbiotas in middle-intestine and sediment. Therefore, our data also suggested that the composition of bacterial community could be shaped in the intestine, and the hind-intestine was more selective in community composition.

At genus level, our data showed that the dominant bacterial taxa were obviously different between CZ and CH groups, while the dominant bacterial taxa were similar between RZ and RH groups (Table S13). These results suggested that the construction of bacterial community was associated with host species. As revealed that *Aeromonas hydrophila* and *Vibrio parahaemolyticus* were the bacterial pathogens for crab^45^. It has been reported that Shigella has expanded from humans to animals^46^, and it is one of the major pathogens that causes diarrheal diseases in humans and animals^47–49^. Previous studies showed that *Bacillus*, *Bifidobacterium*, *Enterococcus* and *Lactobacillus*, as potential probiotics, were beneficial to the health of aquatic animals^50,51^. For example, Zheng et al.^52^ reported that *Lactobacillus plantarum* could improve the growth performance of *Litopenaeus vannamei*. Yang et al.^38^ reported that *Bacillus cereus* was helpful to promote intestinal microbiota homeostasis of *Apostichopus japonicus*. In present study, both of beneficial bacteria and pathogenic bacteria tended to be enriched in the intestine. Thus, we could speculate that this might be related to maintain the health status of crab intestine.

The rare taxa encompass a tremendous species diversity but in low abundance within the community^53^. Some rare taxa may be inactive and even permanently dormant, while others may conditionally bloom under favorable environmental conditions and conduct important eco-processes^53,54^. These characteristics of rare taxa inspired researchers to know them. For example, Sogin et al.^55^ reported that a relatively small number of different populations dominate all samples, but thousands of low-abundance populations account for most of the observed phylogenetic diversity. Galand et al.^56^ found that the taxonomic composition of the rare microbes was similar to the composition of the abundant phylotypes, and the microbial diversity was largely determined by the rare microbes. Dai et al.^53^ reported that rare taxa and conditionally rare taxa were the main taxonomic composition of bacterial community in the sediments of Hangzhou Bay. As revealed in this study, the main taxonomic compositions of bacterial community were rare taxa and conditionally rare taxa in the sediments and the intestinal microbiota of crab, and the main taxonomic composition of rare taxa and conditionally rare taxa were the same with the abundant taxa (Fig. 4).

Intestinal microbiota could be regarded as a complex ecosystem, including species/community functions, community balance and species cooperation/competition. In microbial ecosystem, species interactions are more important to ecosystem functioning than species richness, especially in complex ecosystems^57–59^. The complex microbial ecosystem of the intestinal tract is unevenly influenced by individual taxa within different microbial communities^60^; and interactions between species form the basis for community structure and ecosystem function^61^. Yang et al.^24^ reported that microbial ecological networks in the middle- and hind-intestine were unique, and species interactions had quite different characteristics between two microbial ecological networks. Meanwhile, Faust et al.^62^ reported that the healthy microbiota showed remarkable variability within and among individuals. In the present study, the structure and the properties of microbial ecological networks in the RZ, RH, CZ and CH groups were different, and the competitive interactions predominated these four networks. Yang et al.^24^ showed the different results that competitive interactions predominated in the microbial community of middle-intestine, whereas cooperative interactions were dominant in the hind-intestine. In our previous study, cooperative interactions predominated in the hind-intestine of *Bostrichthys sinensis*^36^. Thus, the microbial ecological network was not only associated with the different intestinal regions, but also remarkable variability among host species.

Species play different roles in the microbial ecological system, they can be divided into four types: peripherals, module hubs, connectors and network hubs^63^. Species serving as peripherals represent specialists, and species serving as module hubs or connectors represent generalists, and network hubs species signify super-generalists, acting as both module hubs, connectors^64^. Previous studies found that most species served as peripherals in the microbial ecological networks of aquatic animal’s intestine^24,36,65,66^. Our data also showed that peripherals species were the most in the crab’s intestine. The loss of peripherals species can not affect the structure property of ecological network, whereas the extinction of species serving as module hubs, connectors or network hubs may induce a sudden collapse of ecological networks^66–68^. In addition, species serving as module hubs, connectors or network hubs play important roles in coherence of module, and play cornerstone roles in the ecological network^58^. In this study, the species serving as module hubs or connectors belonged to Actinobacteria, Bacteroidetes and Firmicutes in the RZ and RH networks, and these phyla dominated in the microbial compositions of the RZ and RH groups. Similar results were observed in the CZ and CH groups, Bacteroidetes, Firmicutes and Proteobacteria played cornerstone roles in the ecological networks of the CZ and CH groups. These results showed that cornerstone species were closely associated with the dominant species in the ecological network/microbial community, and the compositions of cornerstone species were connected with the host species.

The intestinal microbiota was regarded as the second organ or brain, because it had a far greater diversity of genes, repertoire of metabolic and enzymatic capabilities than their host^69,70^. Know the functions of bacterial community would contribute to understand more about animal health. In present study, the COG and KEGG functions were similar between intestinal microbiota and the corresponding sedimental microbes. Fan et al.^21^ also found that COG function classifications of bacterial community in shrimp intestine were similar with sediment. Upon further analysis, we found that the functional structure in bacterial community between crab intestine and sediment were different, and no significant correlation in bacterial functions was observed between crab intestine and sediment. These might be related to microbial host and living environment.

## Materials and methods

### Sample collection

*Metaplax longipes* (mean body weight approximately 4.68 g) and *Helice japonica* (mean body weight approximately 4.07 g) were collected from Beihai Caotou village, Guangxi, China and these two crabs were collected from the same mudflat. Crabs were collected on the 16^th^ Apr. 2020. The eighteen individuals (similar size) of each crab species were selected for collecting the middle- and hind-intestines. The intestinal samples were obtained according to the method of Sun et al.^18^ with some modifications. Prior to dissection, all crabs were euthanized with an overdose of 3-aminobenzoic acid ethyl ester methanesulfonate (Sigma, Neustadt, Germany). The surface of each crab was sterilized with 70% ethanol to reduce contamination, and the middle- and hind-intestine were transferred to 1.5 mL aseptic tube, respectively. The middle-intestine or hind-intestine of three individuals were pooled as one sample. Meanwhile, sediments were collected from mudflat (within 15 cm of the surface mud). The collection method of sediment was as follows: in a 1 × 1 m quadrat, 5 original soil cores (depth: 15 cm; diameter: 4 cm) were collected. Then these 5 original soil cores were pooled as one sample. For each sample, soil was sieved on a 2 mm mesh to remove rocks and roots. After collection, sample was immediately stored at − 80 °C. All samples were divided into six groups as follows:

RZ group: six middle-intestine samples from *H. japonica*;

RH group: six hind-intestine samples from *H. japonica*;

CZ group: six middle-intestine samples from *M. longipes*;

CH group: six hind-intestine samples from *M. longipes*;

RN group: six sediment samples from sediment surrounding *H. japonica*;

CN group: six sediment samples from sediment surrounding *M. longipes*.

### DNA extraction and sequencing

The total DNA of all samples were extracted by using DNeasy® PowerSoil® Kit according to the handbook (QIAGEN, Germany). The concentration of the total DNA was measured by NanoDrop ND-2000 spectrophotometer (Thermo Scientific, Waltham,MA, USA) and the agarose gel electrophoresis was used to detected the quality of the total DNA. The DNA samples were placed in a dry ice box and sent to Shanghai OE Biotech Co., Ltd for Illumina sequencing on the MiSeq platform. The V3 + V4 regions of 16S rDNA were amplified by the barcoded fusion primers of 343F (5′-TACGGRAGGCAGCAG-3′) and 798R (5′-AGGGTATCTAATCCT-3′).

### Bioinformatics analysis

The raw data was processed by QIIME (version 1.8.0) software^71^. The quality of raw data was controlled by Trimmomatic (version 0.35) software and raw sequence with the length < 50 bp would be discarded. The eligible raw sequences would be merged by Flash (version 1.2.11) software. The clean tags with the length long > 200 bp were obtained by split_libraries (version 1.8.0) software. The chimeric sequences were discarded by UCHIME (version 2.4.2) software. The valid tags were clustered into operational taxonomic unit (OTU) at a 97% identify threshold by Vsearch (version 2.4.2) software. Based on the Silva database, the OTUs were annotated and classified by QIIME software.

The α-diversity indices in terms of Chao1, Observed_species, Shannon and Simpson were calculated by using the QIIME software. Permutational multivariate analysis of variances (PERMANOVA), multi-response permutation procedure (MRPP) and analysis of similarity (ANOSIM) were used to test the dissimilarity between two groups based on Bray–Curtis distance methods by using the vegan package in R (v. 3.6.3). The UpSet plot was drew by R with upset package. Procrustes analysis was performed to explore the co-occurrence associations of microbial community between two groups by using the vegan and ggplot2 packages in R. The UPGMA analysis based on the Bray–Curtis dissimilarity was performed with package of vegan and stats in R. Mantel test of bacterial community was analyzed using vegan package in R.

To detect the potential key species, random forests analysis was performed using method of regression to find out the important taxa by R with randomForest package^72^. Further, indicator species were identified based on the normalized abundances of species by R with indicspecies package, and the significant indicator value (IV) index was calculated by the 9,99-permutation test^73^.

Categories of bacterial taxa method was according to the report of Dai et al.^53^. Six categories based on their range of abundance: (1) rare taxa (RT), OTUs with abundance ≤ 0.1% in all samples; (2) abundant taxa (AT), OTUs with abundance ≥ 1% in all samples; (3) moderate taxa (MT), OTUs with abundance between 0.1 and 1% in all samples; (4) conditionally rare taxa (CRT), taxa with abundance below 1% in all samples and ≤ 0.1% in some samples; (5) conditionally abundant taxa (CAT), taxa with abundance greater than 0.1% in all samples and ≥ 1% in some samples but never rare (≤ 0.1%); (6) conditionally rare or abundant taxa (CRAT), taxa with abundance varying from rare (≤ 0.1%) to abundant (≥ 1%). Bacterial functions were predicted by PICRUSt (version 1.1.0), based on valid tags^74^.

### Ecological network analysis of intestinal microbiota of crab

To explore bacterial interactions in the intestinal microbiota of crab, phylogenetic molecular ecological networks (pMENs) were constructed via the random matrix theory-based interface approach in the molecular ecological network analysis pipeline (MENA, http://ieg2.ou.edu/MENA/)^63^. The modules of each ecological network were naturally separated by the fast greedy modularity optimization method^75^. Software Cytoscape (v 3.8.0) was used to visualize the ecological networks of each group; and each node signified an OTU, corresponding to a microbial population; colors of the nodes indicated different major phyla; blue edges indicated a positive interaction between two individual nodes, while red edges indicated a negative interaction. Node degree is the connection strength between two individual nodes. Topological roles of OTUs were identified in the ecological networks according to the studies of Olesen et al.^64^ and Deng et al.^63^. Overall OTUs could be separated into four types: peripherals, connectors, module hubs and network hubs. Species with both a low Zi (value < 2.5) and a low Pi (value < 0.6) were peripheral species, i.e., they had only a few links and almost always only to species within their module; species with either a high value of Zi (≥ 2.5) or Pi (≥ 0.6) were module hubs species or connectors species, they were highly linked to many species within their own module; species with both a high Zi (≥ 2.5) and a high Pi (≥ 0.6) were network hubs species, they acted as both connectors and module hubs.

### Statistical analysis

The differences between groups at phylum and genus levels were analyzed by using Kruskal–Wallis rank sum test. The differences between α-diversity metrics were analyzed by post-hoc Tukey test. A *P* value < 0.05 was considered to be statistically significant.

## Conclusion

In the present study, Firmicutes and Proteobacteria showed significant differences between intestinal microbiota of the two wild crab species and habitat microbes. The diversity in habitat microbes was significantly higher than that in intestinal microbiota of two wild crab species. In contrast, a similar composition and diversity in intestinal microbiota were observed between two wild crab species. Further bacterial community relationships revealed significant correlation in microbial community between middle-intestine of two wild crab species and habitat. Furthermore, network analysis revealed that the network structure of the intestinal microbiota was not only associated with intestinal regions, but also with the crab species. Based on the findings of this study, we gain a comprehensive understanding of the intestinal microbiota of wild aquatic animals and habitat microbes. In the future study, more animals and species will be needed to further determine the relationships between habitat microbes and the intestinal microbiota of wild aquatic animals.

## Supplementary Information

Supplementary Information.

## Data Availability

The raw sequencing data can be obtained from the Sequence Read Archive (SRA) of the NCBI under the accession number PRJNA695532.
