# Supplementary material for: Profiling intestinal microbiota of Metaplax longipes and Helice japonica and their co-occurrence relationships with habitat microbes
Source: Sci Rep. 2021 Oct 27;11:21217. doi: 10.1038/s41598-021-00810-9 (PMC8551266; doi:10.1038/s41598-021-00810-9)
Supplement: Supplementary file 1 — Supplementary Information. [file 41598_2021_810_MOESM1_ESM.pdf]

Profiling intestinal microbiota of *Metaplax longipes* and *Helice japonica* and their co-occurrence relationships with habitat microbes

Haidong Li[1,*], Shanshan Li[1], Shiliang Fan[2], Yan Xu[3], Xiangli Tian[4]

[1]Zhejiang Ocean University, School of Fishery, Zhoushan 316022, China

[2]MNR Key Laboratory of Marine Eco-Environmental Science and Technology, First Institute of Oceanography, Ministry of Natural Resources, Qingdao 266061, China

[3]Guangxi Key Lab of Mangrove Conservation and Utilization, Guangxi Mangrove Research Center, Beihai 536000, China

[4]Key Laboratory of Mariculture, Ministry of Education, Ocean University of China, Qingdao, China

[*] Corresponding author: Haidongli; Email address: lhd0927@126.com

Table S1 Sequencing data in the RZ, RH, CZ, CH, RN and CN groups..3

Table S2 Dissimilarity tests of the microbial community between two groups using ANOSIM, PERMANOVA and MRPP based on the Bray-Curtis distance. 4

Table S3 Dominant composition in all groups at phylum level. 5

Table S4 Dominant genera, beneficial bacteria and pathogenic bacteria in all groups. 6

Table S5 Dominant composition of rare taxa and conditionally rare taxa in bacterial communities of RZ, RH, CZ, CH, CN and RN groups..7

Table S6 Mantel test of bacterial community showed the relationship between two groups............7

Table S7 Topological properties of the empirical phylogenetic molecular ecological networks of intestinal mircrobiota of four groups in response to their associated random networks. 8

Table S8 Composition and topological properties of the ecological network in all groups. 9

Table S9 The OTUs with node degree ≥ 50 in the RZ, RH, CZ and CH networks.........................10

Table S10 Topological roles of OTUs in four networks..12

Table S11 Mantel test indicating the correlations of bacterial community functions between two groups based on COG data..13

Table S12 Mantel analysis showed the relationship between two groups based on KEGG function data. 13

Table S13 The abundant bacterial taxa (relative abundance ≥ 1%) at genus level in RZ, RH, CZ and CH groups. 14

Fig. S1 Non-metric multi-dimensional scaling (NMDS) plot showed bacterial functional community dissimilarities using Bray-Curtis distance Based on COG function data.......................................15

Fig. S2 The KEGG function classification of the six groups. 16

Fig. S3 Non-metric multi-dimensional scaling (NMDS) plot showed bacterial functional community dissimilarities using Bray-Curtis distance Based on KEGG function data. 17

Table S1 Sequencing data in the RZ, RH, CZ, CH, RN and CN groups.

Group	Sample_ID	valid_tags	OTU_counts	valid mean Length
RZ1	RZ1	75424	658	411.61
	RZ2	78956	1311	415.68
	RZ3	73827	1391	413.87
	RZ4	73363	974	413.23
	RZ5	78746	661	411.88
	RZ6	74346	819	415.75
RH	RH1	75896	1559	418.68
	RH2	69891	1172	412.19
	RH3	73530	674	412.94
	RH4	74073	1077	415.12
	RH5	75096	580	415.79
	RH6	71962	790	411.28
CZ	CZ1	75081	547	411.79
	CZ2	74613	598	407.65
	CZ3	75212	550	410.37
	CZ4	72200	600	414.62
	CZ5	73219	605	409.48
	CZ6	74844	797	415.89
CH	CH1	78836	1244	416.71
	CH2	76130	1671	412.49
	CH3	73714	2515	415.94
	CH4	62837	2598	397.84
	CH5	64362	1979	413.73
	CH6	76293	1480	418.18
RN	RN1	79363	4242	419.66

	RN2	80484	4198	420.09
	RN3	79088	4533	419.39
	RN4	84195	3733	420.02
	RN5	80918	4184	419.62
	RN6	81246	4364	419.86
CN	CN1	80185	3912	420.18
	CN2	80471	3856	420.01
	CN3	79416	4122	420.01
	CN4	82533	3988	420.09
	CN5	80447	4274	420.27
	CN6	81827	4030	420.37

Table S2 Dissimilarity tests of the microbial community between two groups using ANOSIM, PERMANOVA and MRPP based on the Bray-Curtis distance.

Group	PERMANOVA		ANOSIM		MRPP	
	R	*P*	F	*P*	A	*P*
CZ vs CH	2.687	0.002	0.924	0.002	0.760	0.004
CZ vs RZ	1.549	0.005	0.448	0.004	0.761	0.003
CZ vs RH	1.458	0.001	0.496	0.007	0.771	0.003
CZ vs CN	9.161	0.005	1.000	0.004	0.603	0.002
CZ vs RN	8.728	0.001	1.000	0.003	0.616	0.004
CH vs RZ	1.834	0.007	0.546	0.009	0.770	0.010
CH vs RH	1.763	0.015	0.494	0.004	0.779	0.013

CH vs CN	7.661	0.004	0.959	0.002	0.612	0.001
CH vs RN	7.272	0.004	0.956	0.002	0.625	0.002
RZ vs RH	1.106	0.201	0.120	0.149	0.780	0.185
RZ vs CN	8.442	0.002	1	0.004	0.612	0.002
RZ vs RN	8.042	0.001	1	0.003	0.625	0.002
RH vs CN	8.083	0.004	1	0.003	0.622	0.001
RH vs RN	7.700	0.002	1	0.003	0.635	0.002
CN vs RN	1.450	0.029	0.381	0.022	0.475	0.027

Table S3 Dominant composition in all groups at phylum level.

	RZ	RH	CZ	CH	CN	RN
Acidobacteria	0.56 ± 0.18^{a}	0.45 ± 0.15^{a}	0.64 ± 0.18^{a}	0.33 ± 0.18a	4.83 ± 0.07^{b}	5.13 ± 0.19^{b}
Actinobacteria	9.70 ± 4.06^{a}	5.74 ± 2.59^{a}	10.15 ± 4.59^{a}	1.80 ± 0.72^{a}	4.56 ± 0.25^{a}	4.46 ± 0.25^{a}
Bacteroidetes	33.29 ± 6.16^{b}	33.20 ± 2.82^{b}	28.89 ± 7.65^{b}	36.33 ± 2.65^{b}	16.68 ± 1.00^{a}	18.66 ± 1.27^{a}
Epsilonbacteraeota	1.29 ± 0.35^{a}	1.62 ± 0.36^{a}	1.50 ± 0.25^{a}	11.60 ± 4.75^{b}	0.49 ± 0.03^{a}	0.27 ± 0.04^{a}
Firmicutes	27.36 ± 3.49^{b}	37.05 ± 4.51^{b}	27.46 ± 2.98^{b}	25.56 ± 7.39^{b}	2.09 ± 0.35^{a}	3.16 ± 0.49^{a}
Gemmatimonadetes	0.88 ± 0.51^{a}	0.43 ± 0.23^{a}	0.26 ± 0.08^{a}	0.38 ± 0.22^{a}	5.37 ± 0.11^{b}	5.68 ± 0.23^{b}
Proteobacteria	21.80 ± 2.65^{b}	14.21 ± 2.70^{ab}	21.19 ± 5.08^{b}	11.18 ± 2.44^{a}	61.10 ± 1.05^{c}	57.42 ± 1.95^{c}
Tenericutes	2.71 ± 1.56^{ab}	4.98 ± 4.24^{ab}	1.19 ± 0.38^{ab}	9.37 ± 4.53^{b}	0.06 ± 0.10^{a}	0.08 ± 0.02^{a}

Table S4 Dominant genera, beneficial bacteria and pathogenic bacteria in all groups.

		RZ	RH	CZ	CH	CN	RN
dominant genera	Arcobacter	0.47 ± 0.42[a]	0.71 ± 0.39[a]	0.12 ± 0.04[a]	11.29 ±4.79[b]	0.00 ± 0.00[a]	0.00 ± 0.00[a]
	Bacteroides	6.42 ± 0.65[a]	13.09 ± 3.51[a]	15.79 ± 7.60[a]	8.76 ± 5.95[a]	5.82 ± 1.26[a]	8.76 ± 1.70[a]
	Bifidobacterium	6.73 ± 4.18[a]	2.99 ± 2.01[a]	7.98 ± 4.69[a]	0.59 ± 0.36[a]	0.03 ± 0.01[a]	0.04 ± 0.01[a]
	Candidatus_Hepatoplasma	1.53 ±1.31[a]	4.73 ± 4.24[a]	0.24 ± 0.15[a]	8.24 ± 4.49[a]	0.00 ± 0.00[a]	0.00 ± 0.00[a]
	Escherichia-Shigella	5.09 ± 2.34[ab]	2.99 ± 1.99[ab]	10.53 ± 5.28[b]	0.35 ± 0.16[a]	0.13 ± 0.03[a]	0.20 ± 0.04[a]
	[Ruminococcus]_gnavus_group	3.80 ± 2.28[a]	1.81 ± 1.42[a]	3.75 ± 2.20[a]	0.12 ± 0.04[a]	0.02 ± 0.00[a]	0.04± 0.02[a]
	Woeseia	0.04 ± 0.02[a]	0.01 ± 0.00[a]	0.00 ± 0.00[a]	0.09 ± 0.08[a]	4.24 ± 0.12[c]	3.67 ± 0.15[b]
beneficial bacteria	Bacillus	0.04 ± 0.03[a]	0.03 ± 0.02[a]	0.02 ± 0.02[a]	0.01 ± 0.01[a]	0.01 ± 0.00[a]	0.01 ± 0.00[a]
	Bifidobacterium	6.73 ± 4.18[a]	2.99 ± 2.01[a]	7.98 ± 4.69[a]	0.59 ± 0.36[a]	0.03 ± 0.01[a]	0.04 ± 0.01[a]
	Enterococcus	0.48 ± 0.13[a]	0.59 ± 0.49[a]	0.25 ± 0.07[a]	1.38 ± 1.18[a]	0.01 ± 0.00[a]	0.04 ± 0.01[a]
	Lactobacillus	1.28 ± 0.30[ab]	2.69 ± 1.35[b]	1.23 ± 0.64[ab]	0.35 ± 0.18[a]	0.02 ± 0.00[a]	0.03 ± 0.01[a]
pathogenic bacteria	Aeromonas	0.01 ± 0.01[a]	0.00 ± 0.00[a]	0.08 ± 0.06[a]	0.00 ± 0.00[a]	0.01 ± 0.01[a]	0.00 ± 0.00[a]
	Desulfovibrio	0.16 ± 0.06[b]	0.10 ± 0.01[a]	0.17 ±0.05[b]	0.17 ± 0.08[b]	0.01 ± 0.00[a]	0.00 ± 0.00[a]
	Escherichia-Shigella	5.09 ± 2.34[ab]	2.99 ± 1.99[ab]	10.53 ± 5.28[b]	0.35 ± 0.16[a]	0.13 ± 0.03[a]	0.20 ± 0.04[a]
	Vibrio	0.13 ± 0.06[a]	0.71 ± 0.49[a]	0.02 ± 0.02[a]	0.53 ± 0.37[a]	0.04 ± 0.01[a]	0.06 ± 0.01[a]

Table S5 Dominant composition of rare taxa and conditionally rare taxa in bacterial communities of RZ, RH, CZ, CH, CN and RN groups.

Categories			RZ	RH	CZ	CH	CN	RN
rare taxa, RT/%	p__Bacteroidetes	c__Bacteroidia	43.74	39.05	25.39	26.95	-	-
	p__Firmicutes	c__Clostridia	20.09	21.23	32.10	29.53	-	-
	p__Proteobacteria	c__Deltaproteobacteria	-	-	-	-	27.64	25.85
	p__Proteobacteria	c__Gammaproteobacteria	-	-	-	-	16.70	16.24
conditionally rare taxa, CRT/%	p__Bacteroidetes	c__Bacteroidia	30.59	27.63	27.73	21.42	-	-
	p__Firmicutes	c__Clostridia	28.23	33.22	35.28	33.99	-	-
	p__Proteobacteria	c__Deltaproteobacteria	-	-	-	-	21.37	23.77
	p__Proteobacteria	c__Gammaproteobacteria	-	-	-	-	15.47	15.79

Note: “-” represented corresponding class that was not dominant class in the corresponding category.

Table S6 Mantel test of bacterial community showed the relationship between two groups.

Group	Spearman	
	r	*P*
CZ vs CN	0.5393	0.0167
CH vs CN	0.1000	0.3486
RZ vs RN	0.5036	0.0472
RH vs RN	-0.2393	0.8347

Table S7 Topological properties of the empirical phylogenetic molecular ecological networks of intestinal mircrobiota of four groups in response to their associated random networks.

Groups	Empirical networks							Random networks[a]		
	Similarity threshold (st)	Network size (n)	R^2 of power law	Average connectivity (*avg*K)	Average path length (GD)[b]	Average clustering coefficient (*avg*CC)	Modularity (Module No.)	Average path length (GD)	Avg. clustering coefficient (*avg*CC)	Modularity (Module No.)
RZ	0.97	487	0.649	13.231	5.831[c]	0.306[d]	0.773(18)[e]	2.810±0.014	0.061±0.004	0.218±0.003
RH	0.97	427	0.566	9.856	6.760[c]	0.354[e]	0.851(36)[e]	2.990±0.018	0.047±0.004	0.265±0.004
CZ	0.97	344	0.359	8.728	8.714[c]	0.264[ef]	0.879(29)[e]	3.071±0.022	0.037±0.003	0.288±0.004
CH	0.97	1036	0.570	18.434	4.911[c]	0.395[df]	0.852(41)[e]	2.868±0.009	0.046±0.003	0.178±0.003

a. Random networks were generated by rewiring all nodes and links corresponding to empirical networks 100 times.

b. GD, geodesic distance.

c. Significant difference ($P<0.01$) in average path between any two groups based on *Z* test.

d. Significant difference ($P<0.01$) in average clustering coefficient between RZ and CH groups based on *Z* test.

e. Significant difference ($P<0.01$) in average clustering coefficient between RH and CZ groups based on *Z* test.

f. Significant difference ($P<0.01$) in average clustering coefficient between CZ and CH groups based on *Z* test.

g. Significant difference ($P<0.01$) in modularity for RZ group with other three groups based on *Z* test.

Table S8 Composition and topological properties of the ecological network in all groups.

Index	RZ	RH	CZ	CH
p__Acidobacteria	3	5	1	9
p__Actinobacteria	29	29	16	45
p__Bacteroidetes	150	115	107	405
p__Chloroflexi	1	0	1	1
p__Cyanobacteria	1	0	0	1
p__Deferribacteres	2	1	1	1
p__Epsilonbacteraeota	5	3	4	13
p__Firmicutes	197	188	150	328
p__Fusobacteria	4	3	3	3
p__Gemmatimonadetes	4	3	0	3
p__Nitrospirae	0	0	0	1
p__Patescibacteria	0	1	1	4
p__Proteobacteria	85	71	54	180
p__Spirochaetes	0	2	0	0
p__Tenericutes	5	6	6	40
p__Verrucomicrobia	1	0	0	1
p__Zixibacteria	0	0	0	1
Total number of OTUs	487	427	344	1036
The number of modules	17	35	28	40
Total number of edges	3235	2119	1523	9761
The number of blue edges	1088	720	461	2836
The number of red edges	2147	1399	1062	6925
The number of module hubs	3	6	1	10
The number of connectors	2	0	0	2

Table S9 The OTUs with node degree ≥ 50 in the RZ, RH, CZ and CH networks.

Group	Name	Node degree	No. module	Zi	Pi	phylum
RZ	OTU9475	50	0	1.95	0	p__Firmicutes
	OTU11611	50	0	1.95	0	p__Firmicutes
RH	OTU11312	53	7	2.62	0.04	p__Actinobacteria
	OTU11744	53	7	2.62	0.04	p__Firmicutes
	OTU2635	52	7	2.62	0.00	p__Firmicutes
	OTU5096	52	7	2.62	0.00	p__Actinobacteria
	OTU11977	51	7	2.45	0.04	p__Firmicutes
	OTU1525	50	7	2.45	0.00	p__Firmicutes
CZ	0	0	0	0	0	0
CH	OTU102	118	0	5.67	0.20	p__Proteobacteria
	OTU8	116	0	5.61	0.19	p__Firmicutes
	OTU567	89	2	4.04	0.50	p__Bacteroidetes
	OTU11	71	0	3.19	0.15	p__Firmicutes
	OTU4604	56	4	1.28	0.00	p__Firmicutes
	OTU212	55	4	1.00	0.07	p__Bacteroidetes
	OTU2304	55	4	1.19	0.00	p__Firmicutes
	OTU5188	55	4	1.19	0.00	p__Firmicutes
	OTU10788	55	4	1.19	0.00	p__Firmicutes
	OTU1947	54	4	1.10	0.00	p__Actinobacteria
	OTU2158	54	4	1.10	0.00	p__Actinobacteria
	OTU2184	54	4	1.10	0.00	p__Actinobacteria
	OTU2759	54	4	1.10	0.00	p__Actinobacteria
	OTU280	54	4	0.82	0.10	p__Bacteroidetes
	OTU10271	54	9	2.10	0.00	p__Bacteroidetes
	OTU11847	54	9	2.10	0.00	p__Bacteroidetes
	OTU641	54	9	2.10	0.00	p__Firmicutes
	OTU2049	54	4	1.10	0.00	p__Firmicutes
	OTU2257	54	4	1.10	0.00	p__Firmicutes
	OTU2526	54	4	1.10	0.00	p__Firmicutes
	OTU3694	54	4	1.10	0.00	p__Firmicutes
	OTU4558	54	4	1.10	0.00	p__Firmicutes
	OTU2111	54	4	1.10	0.00	p__Proteobacteria
	OTU2489	54	4	1.10	0.00	p__Proteobacteria
	OTU5167	54	9	2.10	0.00	p__Proteobacteria
	OTU1927	53	4	1.00	0.00	p__Bacteroidetes
	OTU5542	53	9	1.73	0.14	p__Bacteroidetes
	OTU15660	53	4	1.00	0.00	p__Bacteroidetes
	OTU15915	53	4	1.00	0.00	p__Bacteroidetes
	OTU819	53	10	1.26	0.00	p__Firmicutes

OTU2501	53	4	1.00	0.00	p__Firmicutes
OTU2954	53	4	1.00	0.00	p__Firmicutes
OTU11447	53	4	1.00	0.00	p__Firmicutes
OTU838	52	4	0.35	0.20	p__Bacteroidetes
OTU2033	52	4	0.91	0.00	p__Firmicutes
OTU2115	52	4	0.91	0.00	p__Firmicutes
OTU5333	52	4	0.91	0.00	p__Firmicutes
OTU1901	52	4	0.91	0.00	p__Proteobacteria
OTU167	51	9	1.88	0.00	p__Bacteroidetes
OTU536	51	9	1.88	0.00	p__Bacteroidetes
OTU863	51	9	1.88	0.00	p__Bacteroidetes
OTU12254	51	4	0.63	0.08	p__Bacteroidetes
OTU13267	51	9	1.80	0.04	p__Bacteroidetes
OTU495	51	9	1.88	0.00	p__Firmicutes
OTU1234	51	9	1.80	0.04	p__Firmicutes
OTU4587	51	9	1.88	0.00	p__Firmicutes
OTU1105	51	10	1.07	0.00	p__Proteobacteria
OTU1527	50	4	0.63	0.04	p__Actinobacteria
OTU1013	50	10	0.97	0.00	p__Bacteroidetes
OTU3604	50	10	0.97	0.00	p__Bacteroidetes

Table S10 Topological roles of OTUs in four networks.

Treatment	Topological roles		Module number	Phylogenetic associations
RZ	Module hubs	OTU60	4	p__Bacteroidetes
		OTU267	4	p__Bacteroidetes
		OTU980	5	p__Bacteroidetes
	Connectors	OTU449	2	p__Firmicutes
		OTU12661	5	p__Firmicutes
RH	Module hubs	OTU70	8	p__Firmicutes
		OTU2819	5	p__Bacteroidetes
		OTU2635	7	p__Firmicutes
		OTU5096	7	p__Actinobacteria
		OTU11312	7	p__Actinobacteria
		OTU11744	7	p__Firmicutes
CZ	Module hubs	OTU72	2	p__Firmicutes
CH	Module hubs	OTU8	0	p__Firmicutes
		OTU11	0	p__Firmicutes
		OTU33	5	p__Proteobacteria
		OTU95	13	p__Firmicutes
		OTU102	0	p__Proteobacteria
		OTU141	8	p__Bacteroidetes
		OTU567	2	p__Bacteroidetes
		OTU1595	3	p__Bacteroidetes
		OTU1638	14	p__Bacteroidetes
		OTU2825	2	p__Bacteroidetes
	Connectors	OTU346	0	p__Bacteroidetes
		OTU363	0	p__Proteobacteria

Table S11 Mantel test indicating the correlations of bacterial community functions between two groups based on COG data.

	Pearson		Spearman	
	r	p	r	p
RZ vs RH	0.092	0.324	0.082	0.356
RZ vs CN	-0.132	0.575	-0.121	0.549
RZ vs RN	0.097	0.304	0.025	0.443
RH vs CN	-0.459	0.990	-0.400	0.883
RH vs RN	-0.505	0.944	-0.539	0.947
CZ vs CH	-0.510	0.993	-0.486	0.968
CZ vs CN	0.320	0.204	0.404	0.175
CZ vs RN	-0.149	0.736	-0.008	0.660
CH vs CN	-0.254	0.821	-0.189	0.731
CH vs RN	0.003	0.496	0.061	0.417

Table S12 Mantel analysis showed the relationship between two groups based on KEGG function data.

	Pearson		Spearman	
	r	p	r	p
RZ vs RH	0.092	0.347	0.028	0.413
RZ vs CN	-0.176	0.643	-0.236	0.771
RZ vs RN	0.167	0.233	0.082	0.368
RH vs CN	-0.503	0.994	-0.521	0.934
RH vs RN	-0.494	0.961	-0.514	0.960
CZ vs CH	-0.503	0.986	-0.468	0.965
CZ vs CN	0.289	0.025	0.357	0.156
CZ vs RN	-0.124	0.689	-0.157	0.715
CH vs CN	-0.188	0.696	-0.142	0.647
CH vs RN	0.012	0.481	0.071	0.403

Table S13 The abundant bacterial taxa (relative abundance ≥ 1%) at genus level in RZ, RH, CZ and CH groups.

Index	RZ	RH	CZ	CH
[Anaerorhabdus]_furcosa_group	-	1.61 ± 0.91	-	1.24 ± 0.42
[Ruminococcus]_gnavus_group	3.80 ± 2.28	1.81 ± 1.42	3.75 ± 2.20	-
Acinetobacter	2.98 ± 1.89	-	1.04 ± 0.42	-
Alistipes	1.41 ± 0.37	-	1.20 ± 0.35	-
Arcobacter	-	-	-	11.29 ±4.79
Bacteroides	6.42 ± 0.65	13.09 ± 3.51	15.79 ± 7.60	8.76 ± 5.95
Bifidobacterium	6.73 ± 4.18	2.99 ± 2.01	7.98 ± 4.69	-
Blautia	1.33 ± 0.41	-	-	-
Candidatus_Hepatoplasma	1.53 ±1.31	4.73 ± 4.24	-	8.24 ± 4.49
Clostridium_sensu_stricto_1	1.23 ± 0.99	1.75 ± 0.83	-	-
Dysgonomonas	-	1.10 ± 0.56	-	5.59 ± 1.87
Enterococcus	-	-	-	1.38 ± 1.18
Escherichia-Shigella	5.09 ± 2.34	2.99 ± 1.99	10.53 ± 5.28	-
Faecalibacterium	1.02 ± 0.20	1.93 ± 0.91	2.34 ± 1.22	1.18 ± 0.89
Klebsiella	2.87 ± 1.06	1.98 ± 1.77	2.03 ± 0.79	-
Lachnospiraceae_NK4A136_group	1.36 ± 0.30	1.51 ± 0.49	1.05 ± 0.22	1.45 ± 0.67
Lactobacillus	1.28 ± 0.30	2.69 ± 1.35	1.23 ± 0.64	-
Marinifilum	-	4.04 ± 2.37	-	2.49 ± 0.91
Myroides	1.69 ± 1.65	-	-	-
Parabacteroides	1.09 ± 0.31	-	-	-
Prevotella_9	1.31 ± 0.36	-	1.27 ± 0.37	-
Rikenellaceae_RC9_gut_group	-	-	1.30 ± 0.28	-
Roseburia	-	1.99 ± 1.09	-	-
Ruminococcaceae_UCG-014	-	-	-	1.50 ± 1.27
Ruminococcus_1	-	-	-	1.35 ± 0.95
ZOR0006	-	3.31 ± 2.10	-	3.26 ± 1.03

Note: “-” represented genus that was not abundant bacterial taxa in the group.

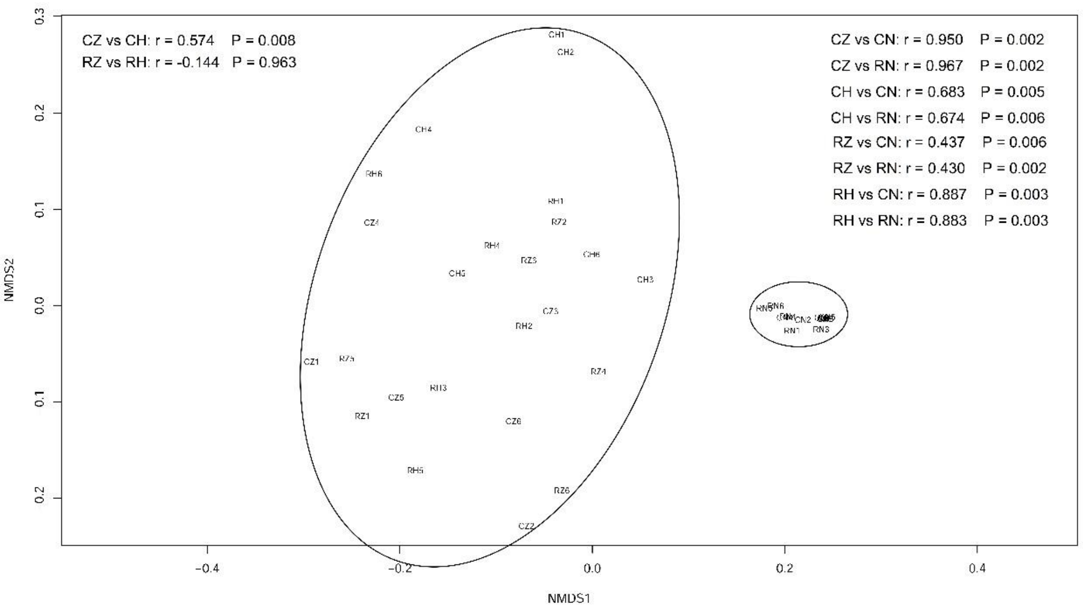

Fig. S1 Non-metric multi-dimensional scaling (NMDS) plot showed bacterial functional community dissimilarities using Bray-Curtis distance Based on COG function data. ANOSIM analysis showed the difference between two groups ($P < 0.05$).

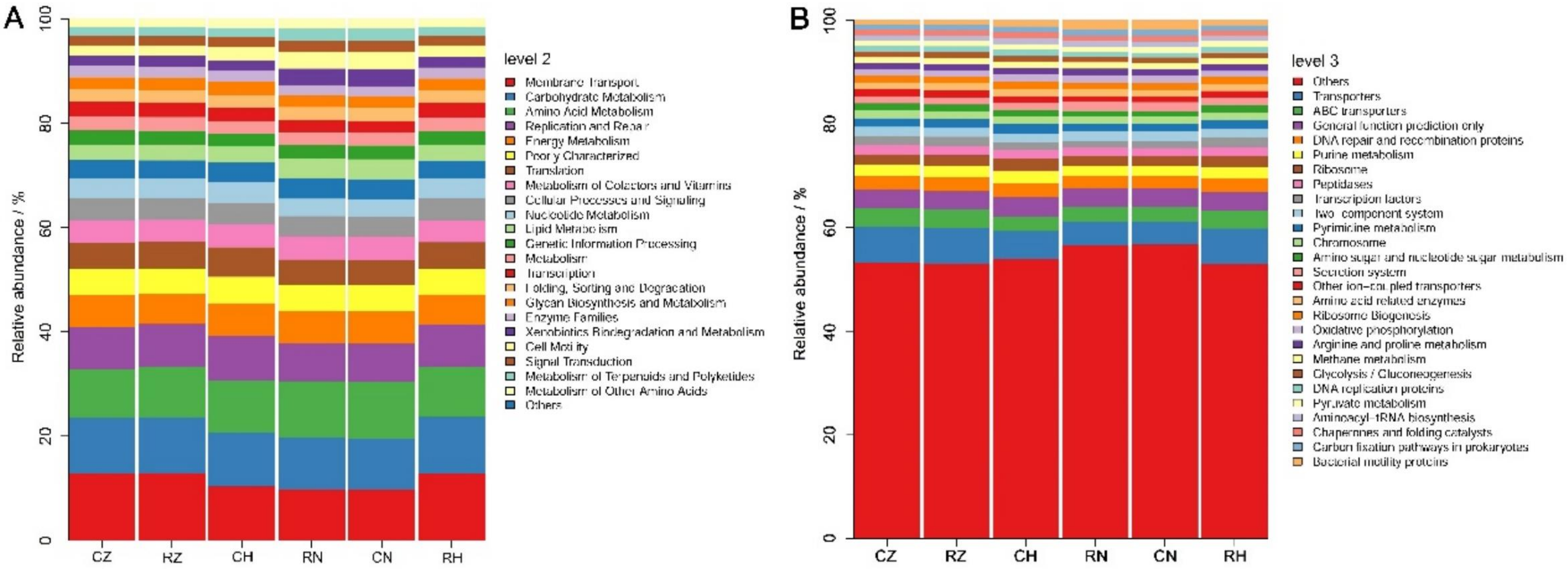

Fig. S2 The KEGG function classification of the six groups.

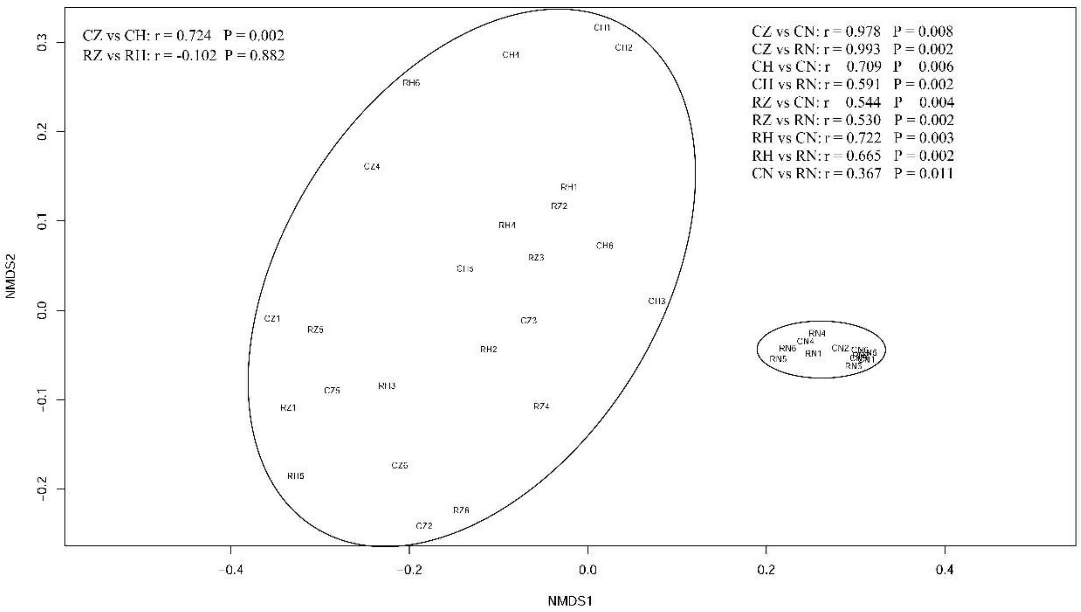

Fig. S3 Non-metric multi-dimensional scaling (NMDS) plot showed bacterial functional community dissimilarities using Bray-Curtis distance Based on KEGG function data. ANOSIM analysis showed the difference between two groups ($P < 0.05$).
